# Draft Genome Sequence of the Ant-Associated Fungus *Phialophora attae* (CBS 131958)

**DOI:** 10.1128/genomeA.01099-15

**Published:** 2015-11-19

**Authors:** Leandro F. Moreno, J. Benjamin Stielow, Michel de Vries, Vinicius A. Weiss, Vania A. Vicente, Sybren de Hoog

**Affiliations:** a CBS, KNAW, Fungal Biodiversity Centre, Utrecht, The Netherlands; b Department of Pathology, Federal University of Paraná, Curitiba, Paraná, Brazil; c Laboratory of Bioinformatics, Federal University of Paraná, Professional and Technological Education Sector, Curitiba, Paraná, Brazil

## Abstract

The black yeast *Phialophora attae* was isolated from the cuticle of tropical ant gynes. The ant-fungus association is sustained due to symbiotic evolutionary adaptations that allow fungal assimilation and tolerance of toxic compounds produced by the ant. The genome sequence of the first ant-associated fungus, *P. attae*, is presented here.

## GENOME ANNOUNCEMENT

Black yeast-like fungi, which are members of the order *Chaetothyriales*, are commonly found to colonize hostile environments. They have a competitive advantage in habitats with a scarcity of nutrients or that are contaminated with toxic hydrocarbons (1); adaptations enhancing the tolerance of extreme conditions allow these fungi to thrive in habitats where eutrophic saprobes are not regularly present (2). For example, black yeasts have been isolated from deserted mine shafts, from industrial biofilters, and repeatedly from ant nests or their exoskeletons (3, 4). Aromatic compounds produced by ants are toxic for most life forms and have antifungal and antibacterial properties (5). Cuticular lipids are involved in chemical communication and colony-mate recognition (6). The ant-fungus symbiosis probably originated 50 million years ago (7, 8). Interestingly, isolates of black yeasts similar to *Phialophora* located in basal and derived ant genera suggest this association might have been started early in the evolutionary history of this symbiosis (9).

*Phialophora attae* CBS 131958 was isolated from the cuticle of gynes of *Atta capiguara* (10). Phylogenetic analyses showed this ant-associated species is affiliated to the europaea clade (*Cyphellophoraceae*), which comprises members involved in human cutaneous and superficial infections and plant debris-inhabiting species (11).

The strain *P. attae* CBS 131958 was cultured in malt extract broth (MEB), with shaking at 150 rpm at 25° for 7 days. DNA was extracted via a cetyltrimethylammonium bromide (CTAB)-based method and phenol-chloroform/isoamyl alcohol. Total DNA was purified with the DNeasy blood and tissue kit (Qiagen). Two hundred-base-read libraries were constructed using NEBNext fast DNA fragmentation and library prep kit for Ion Torrent (Thermo Fisher Scientific). Genomic sequence reads were generated on the Ion Torrent PGM platform (Template OT2 200 kit, Ion Sequencing 200 kit, and Ion Chip kit 318 V2; Thermo Fisher Scientific). The reads were assembled *de novo* using SPAdes version 3.5.0 (12) and Newbler version 2.6 (Roche). The draft comprises 139 contigs, with an *N*_50_ of 959,784 bp. The genome size was estimated to be 30.4 Mb, with a G+C content of 53.56%. Repetitive elements were identified in the assembly using RepeatMasker (http://www.repeatmasker.org) and RepeatModeler (http://www.repeatmasker.org/RepeatModeler.html). Protein-coding genes were predicted with GeneMark-ES (13). Gene product names for 11,853 predicted genes were assigned based on top blast hits against the UniProt Knowledgebase (14) and InterProScan (15) searches. The genome contains 53 tRNAs identified using tRNAscan-SE (16). The prediction of 15 secondary metabolite gene clusters were carried out by means of the antiSMASH Web server (http://antismash.secondarymetabolites.org/).

*P. attae* is the first ant-associated fully sequenced black yeast member. Information about its genome sequence might provide a better understanding of the genomic adaptations made to colonize environments rich in aromatic hydrocarbons.

### Nucleotide sequence accession numbers.

This whole-genome shotgun project has been deposited in DDBJ/ENA/GenBank under the accession no. LFJN00000000. The version described in this paper is the first version, LFJN01000000.
